# Hepatitis E virus seroprevalence among the general population in a livestock-dense area in the Netherlands: a cross-sectional population-based serological survey

**DOI:** 10.1186/s12879-016-2160-4

**Published:** 2017-01-05

**Authors:** Arianne B. van Gageldonk-Lafeber, Wim van der Hoek, Floor Borlée, Dick J. J. Heederik, Sofie H. Mooi, Catharina B. M. Maassen, C. Joris Yzermans, Barry Rockx, Lidwien A. M. Smit, Johan H. J. Reimerink

**Affiliations:** 1 National Institute for Public Health and the Environment (RIVM), Bilthoven, The Netherlands; 2 Institute for Risk Assessment Sciences, IRAS, Utrecht University, Utrecht, The Netherlands; 3 Netherlands Institute for Health Services Research (NIVEL), Utrecht, The Netherlands

**Keywords:** Hepatitis E virus, Environmental exposure, Livestock, Seroprevalence, Zoonoses

## Abstract

**Background:**

Recent serological studies indicate that hepatitis E virus (HEV) is endemic in industrialised countries. The increasing trend in the number of autochthonous cases of HEV genotype 3 in Western European countries, stresses the importance to get insight in the exact routes of exposure. Pigs are the main animal reservoir, and zoonotic food-borne transmission of HEV is proven. However, infected pigs can excrete large amounts of virus via their faeces enabling environmental transmission of HEV to humans. This might pose a risk for of neighbouring residents of livestock farming.

**Methods:**

Within a large study on the health of people living in the vicinity of livestock farming we performed a cross-sectional population-based serological survey among 2,494 non-farming adults from the general population in a livestock-dense area in the south of the Netherlands. Participants completed risk factor questionnaires and blood samples of 2,422 subjects (median age 58 years, range 20–72) were tested for anti-HEV IgG using an enzyme immune assay (Wantai). The aim of this study was to determine the HEV seroprevalence and to assess whether seropositivity in adults was associated with living in the vicinity of pig farms.

**Results:**

The average seroprevalence of HEV was 28.7% (95% CI: 26.9–30.5). Determinants associated with an increased risk for HEV seropositivity were male gender and low level of education. There was a clear trend of increasing prevalence with increasing age (Chi-square test for linear trend, X^2^ = 83.1; *p* < 0.001). A high number of pigs within 1,000 m of the residential address was not a risk factor for seropositivity.

**Conclusions:**

This study confirmed the high HEV seroprevalence (29%) in the general population of the Netherlands, but presence of antibodies was not associated with residential proximity to pig farms. The prevalence increased with age from 10% in adolescents to 33% among those aged 50 and above, supporting the assumption of a cumulative lifetime exposure to HEV in the Netherlands as well as a higher infection pressure in the past. Our findings cannot refute the assumption that transmission is primarily food-borne.

## Background

Hepatitis E virus (HEV) is a major cause of enterically transmitted hepatitis, especially in developing countries. However, recent studies show that autochthonous HEV infections are an increasing public health concern in industrialised countries [1–12].

The most common clinical presentation of HEV infection is acute hepatitis. Usually this is a self-limiting disease, with jaundice as the most common symptom [4, 13–15]. In patients with pre-existing liver disease and immunosuppressed patients, the clinical course of disease is often more severe and in developing countries excess mortality is seen in pregnant women [8, 10, 14–16]. However, most HEV infections in industrialised countries are either unrecognised or asymptomatic [1, 14, 16, 17]. This is supported by recent serological studies in several European countries, showing that the anti-HEV IgG seroprevalence in healthy blood donors varied between 7 and 52%, depending on both geographical area and the antibody assay used [17–22].

HEV is a non-enveloped single-stranded RNA virus belonging to the *Hepeviridae* family [16, 17, 23]. Four major genotypes, within species *Orthohepevirus A,* can infect humans [10, 15, 23, 24]. The genotypes 1 and 2 are restricted to human beings, and the genotypes 3 and 4 can infect both human beings and mammals. HEV-1 and HEV-2 are endemic in developing countries, leading to sporadic cases as well as large outbreaks. HEV-3 and HEV-4 are responsible for an increasing number of autochthonous hepatitis E infections worldwide [4, 10, 16, 17, 25]. The more recently discovered HEV genotypes 5 and 6 are so far only detected in animals, while genotype 7 is also described in humans [10, 26–29].

In contrast to HEV-1 and HEV-2, for which the faecal-oral transmission route via contaminated water has been confirmed, the transmission routes of HEV-3 and HEV-4 are largely unclear and the exact source of infection remains unknown for the majority of patients [4, 17, 27, 28]. The increasing trend in the number of autochthonous cases in Western European countries stresses the importance to get insight in the route of infection, enabling the implementation of control measures against human HEV infections [27, 28, 30].

It is hypothesized that HEV-3 and HEV-4 have a zoonotic origin, supported by the high prevalence of HEV-3 and HEV-4 among domestic pigs and wild boars [23, 26, 31]. In the Netherlands, the prevalence rate of HEV-3 in the domestic pig population is estimated at 55%, and domestic pigs may therefore be an important reservoir for human HEV infections [28, 32]. Furthermore, the frequent detection of HEV in pork products and the high similarity between porcine and human HEV sequences suggest zoonotic transmission of HEV-3 and HEV-4 worldwide [33–36]. Zoonotic food-borne transmission has been proven by the identification of identical nucleotide sequences in autochthonous HEV patients and in leftover portions of consumed contaminated food [37–40]. But zoonotic HEV transmission might also occur through exposure to contaminated environments. Infected pigs, which are generally asymptomatic, can excrete large amounts of virus via their faeces in the environment. This may lead to human infections [26–28, 41]. The high anti-HEV serum antibody rates in humans with occupational contact with pigs, like farmers and veterinarians, provide indirect evidence for this route of transmission [42–47]. However, environmental exposure to HEV might also pose a risk for of neighbouring residents of livestock farming, as was the case during the major Dutch Q-fever outbreak in 2007–2010 [48].

In the Netherlands there is an ongoing debate on the environmental health risks as a result of (intensive) livestock farming in areas highly populated with both livestock and people. At a surface of approximately 34.000 km^2^ almost 17 million people are living together with 75 million chickens, 7 million pigs, 4 million cattle and 1.5 million goats and sheep. Within a large study on the health of people living in the vicinity of livestock farming, we performed a serological survey in order to assess the HEV seroprevalence among non-farming adults from the general population in a livestock-dense area in the south of the Netherlands. Furthermore, we investigated whether living close to pig farms is a risk factor for HEV seropositivity, focusing on the role of environmental transmission of HEV from pig farms to residents living in the vicinity of these farms.

## Methods

In 2014 the Livestock Farming and Neighbouring Residents’ Health study (Dutch acronym: VGO) started, aiming to assess the relationship between livestock farming and a number of health outcomes in people living in the eastern part of the province of Noord-Brabant and the northern part of the province of Limburg. This part of the Netherlands is a relatively highly populated rural area with a high density of livestock farms. Within the VGO study a cross-sectional health study was performed, investigating three categories of health effects: 1) respiratory health effects, 2) livestock-associated infections, and 3) carriage of resistant microorganisms. Furthermore, air sampling was conducted in order to assess to what extent residents living in the vicinity of livestock farms were exposed to emissions of these farms, including endotoxin amount, PM10 and DNA of *Staphylococcus aureus* and *E. coli*. Participants of the cross-sectional health study provided blood samples in order to assess (previous) exposure to several livestock-farming associated pathogens, including HEV.

### Study population

**Fig. 1:**
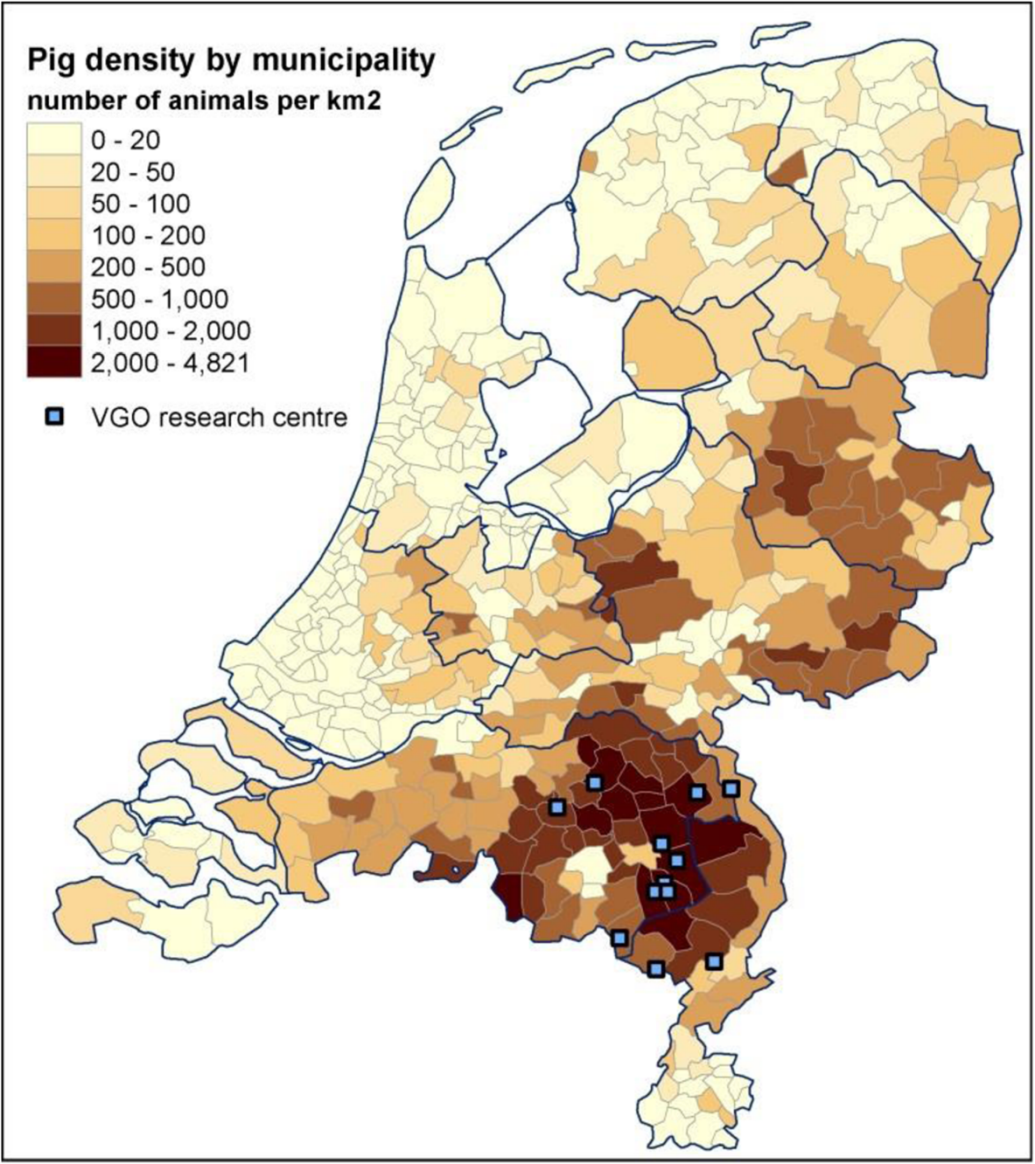
Location of temporary study centres in relation to pig density in the Netherlands in 2014 (Source: Statistics Netherlands)

The study population was selected in a two-step procedure. First, a questionnaire survey was conducted among 14,163 unrelated adults from the general population (aged 18–70 years) living in the study area. Participants were recruited via their general practitioner (GP). In the Netherlands each individual is registered with just one general practice, mostly close to residential address. Pre-defined inclusion criteria have been described previously by Borlée et al. [49]. Second, questionnaire participants who gave consent to be contacted for further studies, and who were not working or living on a farm, were eligible for the serological survey (*n* = 8,714) [50]. Based on their home addresses, twelve temporary study centres were established. Figure 1 shows the location of the temporary study centres, and pig density in the Netherlands in 2014. All participants living within a distance of approximately 10 km of a temporary study centre (*n* = 7,180) were invited to the nearest centre for a medical examination, including blood sampling for serological analyses against livestock-associated pathogens. Furthermore, they were asked to fill in a questionnaire comprising items on symptoms and diseases, home characteristics, smoking habits, education, profession, leisure activities, dietary habits (including the consumption of pork meat), and animal contact, including exposure to animal farm environment during childhood. Data were collected between 10 March 2014 and 27 February 2015. Patients’ privacy was ensured as described earlier [49–51]. In short, medical information and address records were kept separated at all times by using a Trusted Third Party (Stichting Informatie Voorziening Zorg, Houten). The VGO study protocol was approved by the Medical Ethical Committee of the University Medical Centre Utrecht. All participants signed informed consent.

### Exposure from livestock farms

Based on the home address of each participant, several exposure variables were computed, including distance to the nearest pig farm with more than 25 pigs and the total number of pigs within 1,000 m [52]. Information on farm characteristics in the study area was derived from the provincial database of mandatory environmental licenses for keeping livestock for 2012. This database contains amongst others data on number and type of animals and geographic coordinates of farms (specific for type of livestock).

### Serological analyses

Sera were analysed at the National Institute for Public Health and the Environment (RIVM) for the presence of IgG antibodies against HEV, using a commercial anti-HEV IgG enzyme immune assay (Wantai Biological Pharmacy Enterprise Co,. Ltd., Beijing, China). The test was performed according to the instructions of the manufacturer. Samples with anti-HEV IgG ratio <0.90 were considered negative, those with ratio > =0.90 but <1.10 were defined as borderline, and samples with ratio > =1.10 were considered as positive.

### Statistical analyses

Seroprevalence, defined as the percentage of participants with borderline or positive anti-HEV IgG ratio, and 95% confidence intervals (CI) were calculated for the overall study population, per age group and per study centre.

Logistic regression analysis was performed to study potential determinants for HEV seropositivity. The following variables were included in the univariate analysis: age, gender, educational level, country of birth, consumption of pork meat, smoking status, spending childhood in the study region or on a (pig) farm, performing jobs on a farm during childhood, contact with animals during work or study, keeping farm animals and/or pigs for a hobby in the last 5 years, spending time abroad during the last 12 months, visiting a farm in the last 12 months (with or without contact with pigs during that visit) and the numbers of pigs within 1.000 m of the residential address of the participants. All variables with a *P*-value < =0.2 in the univariate analyses were included in the multivariate logistic regression model. Adjusted odds ratios (ORs) and 95% CIs were calculated and a *P*-value <0.05 was used to determine significance. T rend in seroprevalence with increasing age was tested with the Mantel-Haenszel chi square for linear trend.

Median distance from home address to nearest farm with more than 25 pigs was compared between participants with positive or borderline and those with negative anti-HEV IgG ratio using Mann-Whitney U test. This analysis was stratified by six age groups: 20–29, 30–39, 40–49, 50–59, 60–69 and 70 years and older.

The random forest method *(46)* was used to determine the ability of the potential risk factors to predict the anti-HEV IgG ratio or the infection status. Unlike regression, the random forest requires no underlying assumptions about the functional form of the model or distribution of the data and provides a reliable assessment of the contribution of the predictor variables to generate predictions of an outcome. The algorithm predicts the outcome (e.g. anti-HEV IgG ratio) of an individual based upon the individual’s predictor variables, quantifies the prediction accuracy by means of estimates such as the mean square error (or proportion of correct classifications/statuses when predicting the status of an individual) and proportion of explained variance, and ranks the predictor variables according to their relative importance. Relative importance of a predictor variable is calculated by randomly permuting the data for that variable (leaving all other data unchanged) and comparing the prediction accuracy obtained with the ‘shuffled’ data set with that of the original data set. If the variable has little bearing on prediction, the mean square error will remain about the same after shuffling, while if the variable is useful for prediction the mean square error will deteriorate. The increase in mean square error that results from the shuffling is then a measure of variable importance which is used to rank the predictor variables.

Analyses were performed using SAS 9.3 and R version 3.1.0 (Random forest).

## Results

A total of 2,494 of the 7,180 invited persons participated in the cross sectional serological survey, (response rate = 34.7%). Serum samples were available for 2,422 participants (97.1%). The primary reason for missing serum-samples was failure to collect enough blood for analysis. The median age of the participants, from whom a sample was available, was 58 years (range: 20–72 years) and 45.6% was male (Table 1). Although there were some differences between responders and non-responders, a non-response analysis within the initial questionnaire survey comparing the electronical medical records of the subjects recruited via GPs, showed that these differences do not affect the relationship between health and the presence of livestock [53]. A total of 2,163 of the 2,422 participants (89.3%) resided in the province of Brabant and Limburg during childhood, of which 1,816 (84.0%) lived in the specific study region. On average, the participants have lived at their current residential address for 20 years (standard deviation 13.5 years). The number of pigs within 1,000 m of the residential address varied between 0 and 79,057 (median = 2,701; interquartile range: 336–9,035) and the distance to the nearest pig farm with more than 25 pigs ranged from 11 to 2500 m (median = 687 m; interquartile range: 462–932).

**Table 1:** Density of pigs, details of study population and hepatitis E virus seroprevalence by study centre

Study centre	Density of pigs^a^	Number of participants	Median age (years)	Range (years)	Male participants (%)	Seroprevalence^b^ (%)	[95% CI]^c^
Afferden	717	49	59.2	[33.5–71.1]	57.5	25.5	[13.1–38.0]
Asten^d^	3,535	291	61.4	[20.4–71.4]	45.8	34.0	[28.6–39.5]
Bakel	3,756	315	59.3	[21.5–71.7]	47.1	29.2	[24.1–34.3]
Boxtel	988	170	59.3	[24.8–71.6]	45.0	27.2	[20.5–33.9]
Budel	1,080	200	61.4	[21.4–71.6]	49.5	32.6	[25.8–39.4]
Deurne	3,882	132	60.5	[29.0–71.7]	50.0	18.6	[11.7–25.4]
Heeswijk-Dinther	3,812	375	57.5	[20.1–71.4]	37.1	30.1	[25.5–34.8]
Heusden^d^	3,535	72	58.9	[24.2–71.4]	55.6	16.7	[8.1–25.3]
Horn	1,268	85	57.1	[24.0–71.7]	45.2	35.7	[25.5–46.0]
Someren	3,447	170	57.9	[28.6–71.2]	48.2	29.4	[22.6–36.3]
St. Anthonis	4,460	399	57.0	[22.6–71.8]	44.5	26.9	[22.4–31.4]
Stramproy	878	236	57.4	[21.3–72.0]	46.3	26.6	[20.9–32.4]
Total	2,506	2,494	58.6	[20.1–72.0]	45.6	28.7	[26.9–30.5]

^a^Number of pigs per km^2^ by municipality, based on the provincial databases of mandatory environmental licenses for keeping livestock for 2012

^b^Anti-HEV IgG ratio > = 0.90

^c^95% Confidence Interval

^d^Asten and Heusden are both situated in the municipality Asten, and therefore have the same pig density

**Fig. 2:**
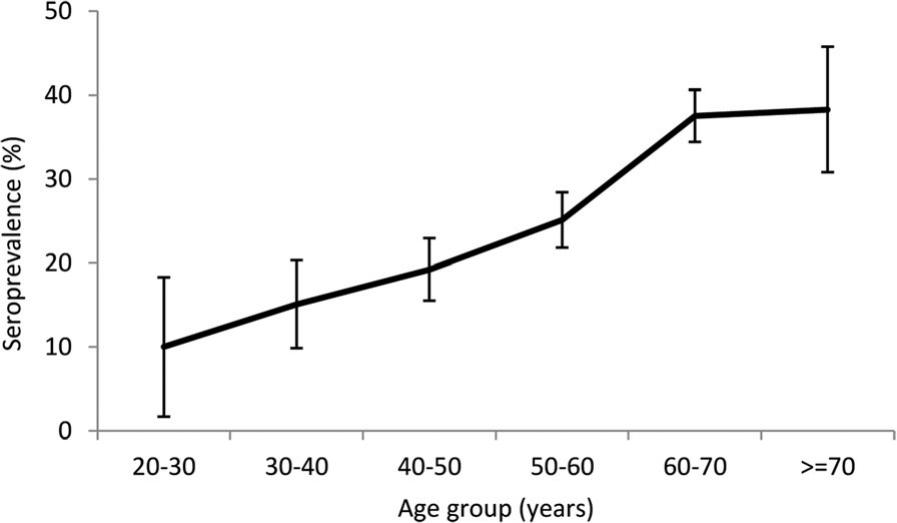
Seroprevalence of antibodies against hepatitis E virus by age group

Positive and borderline positive anti-HEV IgG ratio was found for respectively 666 (27.5%) and 29 (1.2%) of the 2,422 participants. Overall seroprevalence was 28.7% (95% CI: 26.9–30.5; Table 1), ranging between study centres from 16.7% (95% CI: 8.1–25.3) to 35.7% (95% CI: 25.5–46.0). Seroprevalence increased with age (Fig. 2).

Multivariate logistic regression analysis showed an increased risk for HEV seropositivity in participants aged 50 years or older, male participants and participants who reported a low level of education (Table 2). There was a clear trend of increasing prevalence with increasing age (Chi-square test for linear trend, X^2^ = 83.1; *p* < 0.001). A high number of pigs within 1,000 m of residential address was not a risk factor. Other variables for exposure to pigs (e.g. number of pig farms or the presence of at least one pig farm within 1,000 m of residential address) gave similar results in both univariate and multivariate logistic regression analyses as number of pigs within 1,000 m of residential address (data not shown). Participants who reported to follow a diet without pork meat (*n* = 47) did not have a significantly lower risk for HEV seropositivity. Exclusion of the 29 samples with borderline positive anti-HEV IgG ratio did not affect the results of the of univariate and multivariate logistic regression analyses. This also holds when the borderline samples were considered as negative anti-HEV IgG ratio (data not shown).

**Table 2:** Hepatitis E virus seroprevalence in the general population and potential determinants for HEV seropositivity

Determinants	N	Seroprevalence (%)^a^	Crude OR^b^	[95% CI]^c^	Adjusted OR^b,d,e^	[95% CI]^c^
Age (years)						
20–30	50	10.0	Ref.		Ref.	
30–40	179	15.1	1.60	[0.58–4.39]	1.49	[0.54–4.13]
40–50	432	19.2	2.14	[0.82–5.55]	2.01	[0.77–5.26]
50–60	661	25.1	3.02	[1.18–7.72]	2.58	[1.00–6.70]
60–70	938	37.5	5.40	[2.13–13.73]	4.22	[1.63–10.91]
> =70	162	38.3	5.58	[2.10–14.80]	4.15	[1.53–11.24]
Gender						
Female	1,318	25.6	Ref.		Ref.	
Male	1,104	32.3	1.39	[1.16–1.65]	1.21	[1.00–1.47]
Educational level						
High	733	24.8	Ref.		Ref.	
Medium	1,080	26.9	1.12	[0.90–1.38]	1.10	[0.87–1.38]
Low	609	36.5	1.74	[1.37–2.20]	1.36	[1.04–1.76]
Diet without pork meat						
No	2,360	28.8	Ref.		Ref.	
Yes	47	19.2	0.59	[0.28–1.22]	0.72	[0.33–1.59]
*Missing*	*15*					
Ever smoked						
No	1,034	26.5	Ref.		Ref.	
Yes	1,388	30.3	1.21	[1.01–1.45]	0.96	[0.79–1.17]
Childhood in study region						
No	580	32.2	Ref.		Ref.	
Yes	1,816	27.4	0.79	[0.65–0.97]	0.85	[0.68–1.07]
*Missing*	*26*					
Childhood on pig farm						
No	1,928	27.6	Ref.		Ref.	
Yes	468	32.5	1.26	[1.02–1.57]	1.21	[0.94–1.55]
*Missing*	*26*					
Performed jobs on farm during childhood						
No	1,090	26.9	Ref.	-	Ref.	
Yes	1,220	29.7	1.15	[0.96–1.38]	1.02	[0.83–1.27]
*Missing*	*112*					
Number of pigs within 1000 m of residential address (tertiles)						
Low^f^	807	32.1	Ref.		Ref.	
Intermediate^g^	808	26.9	0.78	[0.63–0.96]	0.79	[0.63–0.99]
High^h^	807	27.1	0.79	[0.64–0.98]	0.83	[0.66–1.04]

^a^Including both positive and borderline samples (anti-HEV IgG ratio > = 0.90)

^b^Odds Ratio

^c^95% Confidence Interval

^d^ORs are adjusted for age, gender, educational level, diet without pork meat, ever smoked, childhood in study region, childhood on pig farm, jobs on farm during childhood and number of pigs within 1000 m of residential address

^e^Adjusted ORs and 95% CIs were calculated for cases without missing answers (*N* = 2,301)

^f^Number of pigs within 1000 m < = 1,003

^g^Number of pigs within 1000 m > 1,003 and < = 5,771

^h^Number of pigs within 1000 m > 5,771

Generally, seronegative participants lived closer to pig farms than seropositive participants. This difference was statistically significant for the overall group and for the age group 60–70 years (Table 3).

**Table 3:** Median distance from home address to nearest pig farm by anti-hepatitis E virus IgG ratio

	Positive or borderline anti-HEV IgG ratio^a^			Negative anti-HEV IgG ratio^b^			
Age group (years)	n	Median distance (m)	Range in distance (m)^c^	n	Median distance (m)	Range in distance (m)^c^	*P*-value
20–30	5	689	[469–850]	45	652	[489–843]	1.00
30–40	27	751	[373–1102]	152	581	[440–872]	0.16
40–50	83	702	[416–965]	349	665	[441–900]	0.68
50–60	166	701	[498–955]	495	674	[455–901]	0.28
60–70	352	748	[517–970]	586	686	[430–938]	0.02
> = 70	62	794	[539–1041]	100	672	[528–1004]	0.36
Total	695	730	[509–969]	1727	663	[447–916]	0.001

^a^Anti-HEV IgG ratio > =0.90

^b^Anti-HEV IgG ratio <0.90

^c^Range from the 25th to the 75th percentile of the distance from home address to nearest pig farm

Despite the associations detected and reported above, the random forest method showed that the potential risk factors considered in this study are not at all able to predict anti-HEV IgG ratio nor disease status (results are omitted). Indeed, the proportion of explained variance was practically zero in our prediction analysis, which in particular prevents us from ranking the risk factors.

## Discussion

The present population-based serological survey confirms the high HEV seroprevalence (29%) in the general population of the Netherlands, but provides no evidence for environmental transmission from pig farms to humans living in the vicinity of these farms.

Comparison of the seroprevalence found in this study with results from other European countries is difficult because of considerable differences in the studied populations, as well as the used assay for HEV antibody testing [54–56]. Recent studies using sensitive assays (like the Wantai assay we used) reported HEV seroprevalence varying between 16% and 52% [18, 57–61]. Our seroprevalence (29.7%) corresponds well with the 27% prevalence found in a large serological screening of Dutch blood donors [18]. Both studies used the Wantai assay to measure HEV specific IgG antibodies. In contrast, a population-based seroprevalence study in the general population in the Netherlands in 2006–2007, using a less sensitive assay for detection of prolonged IgG antibodies (MP Diagnostics, France), found a seroprevalence of only 3% [62]. Comparative studies suggest that the Wantai assay gives the most reliable estimate of the HEV seroprevalence, because it is more sensitive, it is positive in a higher proportion of proven infections and it remains positive for longer period post infection compared with the MP assay [56, 58, 59, 63].

In line with other serological studies, we found statistically significant higher seroprevalence in participants aged 50 years and older compared to 20–30 year olds [18, 44, 57, 62, 64]. This higher seroprevalence in older persons might be a result of age-dependent cumulative exposure, but can also be indicative of an age-cohort because of higher pressure of infection in the past. This age-cohort effect is also demonstrated in Denmark, the United Kingdom and the United States [60, 61, 65]. The HEV seroprevalence in blood donors as well as the high number of HEV RNA-positive donors found in serological screenings suggest an increasing HEV incidence in recent years in the Netherlands [18, 66, 67].

Proximity to pig farms and number of animals close to the home were used as proxy of potential environmental exposure to HEV. The lack of a positive association suggests that airborne spread of HEV is unlikely. Other environmental transmission routes cannot be excluded, for example through manure that may be transported to distant places or through surface water. In the Netherlands, HEV RNA has been detected in surface water samples [68]. Furthermore, a recent cross-sectional study suggests that consumption of tap water in France might be a risk factor for HEV infection [69]. Occupational exposure to pigs, which is a potential risk factor for seropositivity, was not investigated in our study since persons living or working on a farm were excluded [44, 47, 70]. Moreover, the present study was not designed to investigate the role of food-borne transmission. Presence of IgG antibodies is an indicator for HEV infection in the past and therefore suited to study exposure to HEV through the environment. Although we found no decreased risk for HEV seropositivity in participants who did not consume pork meat, this finding must be interpreted with caution because of the small numbers of participants on a diet without pork meat (*n* = 47, 2.0%). Moreover, some of these participants might have consumed pork meat in the past. A case-control study focussing mainly on food-borne transmission among patients with acute hepatitis E infection in the Netherlands is ongoing (study period: 2015–2017).

## Conclusions

In conclusion, our study showed that the HEV seroprevalence in the general population in a livestock-dense area in the south of the Netherlands was not associated with living in proximity to pig farms. The higher seroprevalence among older participants supports the assumption of cumulative lifetime exposure to hepatitis E virus in the Netherlands, as well as an age-cohort because of higher pressure of infection in the past. Although it seems plausible that transmission is primarily food-borne, further research is needed to elucidate the exact sources and routes of infection.

## Abbreviations

CI: Confidence interval
GP: General practitioner
HEV: Hepatitis E virus
OR: Odds ratio
RIVM: National Institute for Public Health and the Environment (Dutch acronym)
VGO: Livestock farming and neighbouring residents’ health study (Dutch acronym)
